# Characterization and Comparative Analysis of Small RNAs in Three Small RNA Libraries of the Brown Planthopper (*Nilaparvata lugens*)

**DOI:** 10.1371/journal.pone.0032860

**Published:** 2012-03-06

**Authors:** Qiuhong Chen, Lin Lu, Hongxia Hua, Fei Zhou, Liaoxun Lu, Yongjun Lin

**Affiliations:** 1 National Key Laboratory of Crop Genetic Improvement and National Centre of Plant Gene Research, Huazhong Agricultural University, Wuhan, China; 2 Hubei Insect Resources Utilization and Sustainable Pest Management Key Laboratory, Huazhong Agricultural University, Wuhan, China; University of Illinois-Chicago, United States of America

## Abstract

**Background:**

The brown planthopper (BPH), *Nilaparvata lugens* (Stå;l), which belongs to Homopteran, Delphacidae, is one of the most serious and destructive pests of rice. Feeding BPH with homologous dsRNA in vitro can lead to the death of BPH, which gives a valuable clue to the prevention and control of this pest, however, we know little about its small RNA world.

**Methodology/Principal Findings:**

Small RNA libraries for three developmental stages of BPH (CX-male adult, CC-female adult, CY-last instar female nymph) had been constructed and sequenced. It revealed a prolific small RNA world of BPH. We obtained a final list of 452 (CX), 430 (CC), and 381 (CY) conserved microRNAs (miRNAs), respectively, as well as a total of 71 new miRNAs in the three libraries. All the miRNAs had their own expression profiles in the three libraries. The phylogenic evolution of the miRNA families in BPH was consistent with other species. The new miRNA sequences demonstrated some base biases.

**Conclusion:**

Our study discovered a large number of small RNAs through deep sequencing of three small RNA libraries of BPH. Many animal-conserved miRNA families as well as some novel miRNAs have been detected in our libraries. This is the first achievement to discover the small RNA world of BPH. A lot of new valuable information about BPH small RNAs has been revealed which was helpful for studying insect molecular biology and insect resistant research.

## Introduction

Small RNAs caused a radical revolution in biology in recent years. As there is increasingly more research on small RNAs,the use of small RNA was also increased, especially in the field of RNA interference (RNAi) [1], [2], [3], [4]. These 18 to 30 nucleotide-long sequences regulate various biological processes, often by interfering with mRNA degradation and hindering translation, or causing epigenetic phenomena [5]. They are not translated into proteins and have different forms, of which the three best-understood classes are microRNAs (miRNAs), small interfering RNAs (siRNAs) and piwi-associated RNAs (piRNAs). MicroRNAs (miRNAs) which are different from other small RNAs [6], as their name suggested, are tiny RNA molecules. They are involved in regulating cell death and proliferation, fat metabolism and the differentiation of hematopoietic families in animal. In human, many miRNAs regulate thousands of target genes in a complex network, and miRNAs can control the development of leaves and flowers of plant as well [7], [8], [9]. Endogenous siRNAs, the indispensable parts of living bodies, were also found in animals and plants in recent years. They are often generated from the transposable elements (TE) or the complementary RNA strands from the transcriptions of sense and antisense chains [10], [11], [12], [13]. PiRNA, which was first isolated from animal germline cells, is a class of relatively longer non-coding small RNA and must combine with piwi protein family members to function [14], [15], [16].

Insects are a group of living creatures which have the greatest variety and the most widely distribution of species in the world. Though insect small RNA research is somewhat backward to mammal, nematodes and plants, many small RNAs have been identified in insects whose whole genome sequences have been sequenced, including the fruit fly [17], silkworm [18], bee [19], red flour beetle [20], mosquito [21], and aphid [22], also in insects whose genome sequences haven't been obtained, such as locust [23]. The information of all the small RNAs gave a strong foundation for the research of insect growth and development. The brown planthopper is one of the most serious pests of rice in both temperate and tropical regions of Southeast Asia and has become especially problematic over the past few years. As a long-distance migratory and explosive homopteran pests, BPH has a sucking mouthpart and causes great loss of rice yields by ingesting assimilates from the plant phloem [24]. Feeding BPH with homologous dsRNA in vitro can lead to the interference of relative endogenous genes and cause the death of BPH [25], [26]. This observation gave a valuable clue to the prevention and control work of BPH. However, we know little about the small RNA world of BPH. In this study, three small RNA libraries of developmental stages (CY (the last instar female nymph), CX (male adult), and CC (female adult)) of BPH have been constructed and sequenced. The results demonstrated that the small RNA repertoire in BPH is very rich. These small RNAs have an important guiding role in utilizing RNA interference (RNAi) technology to prevent BPH.

## Results

### Characteristic description and public sequence analysis of the small RNAs

**Figure 1:**
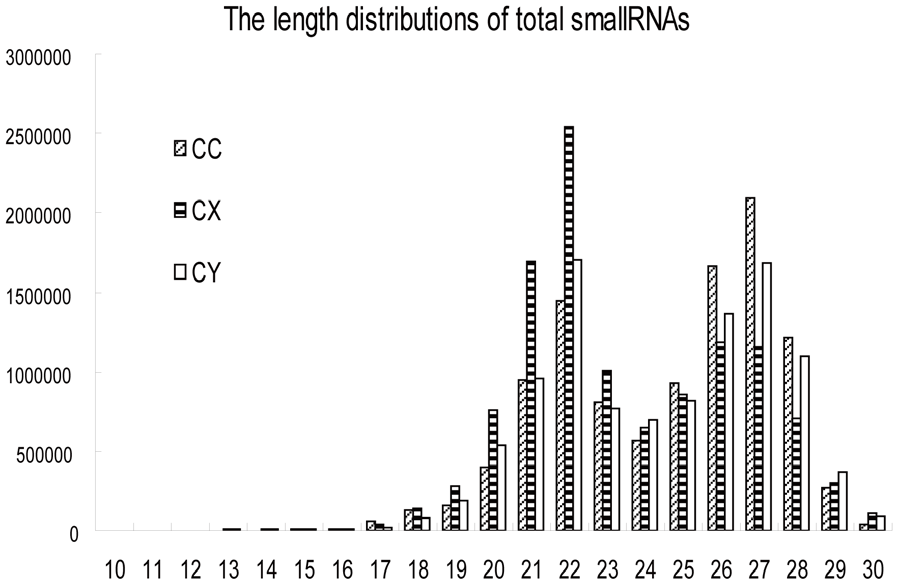
Total small RNA reads length distributions of the three libraries (CC, CY, and CX).

**Figure 2:**
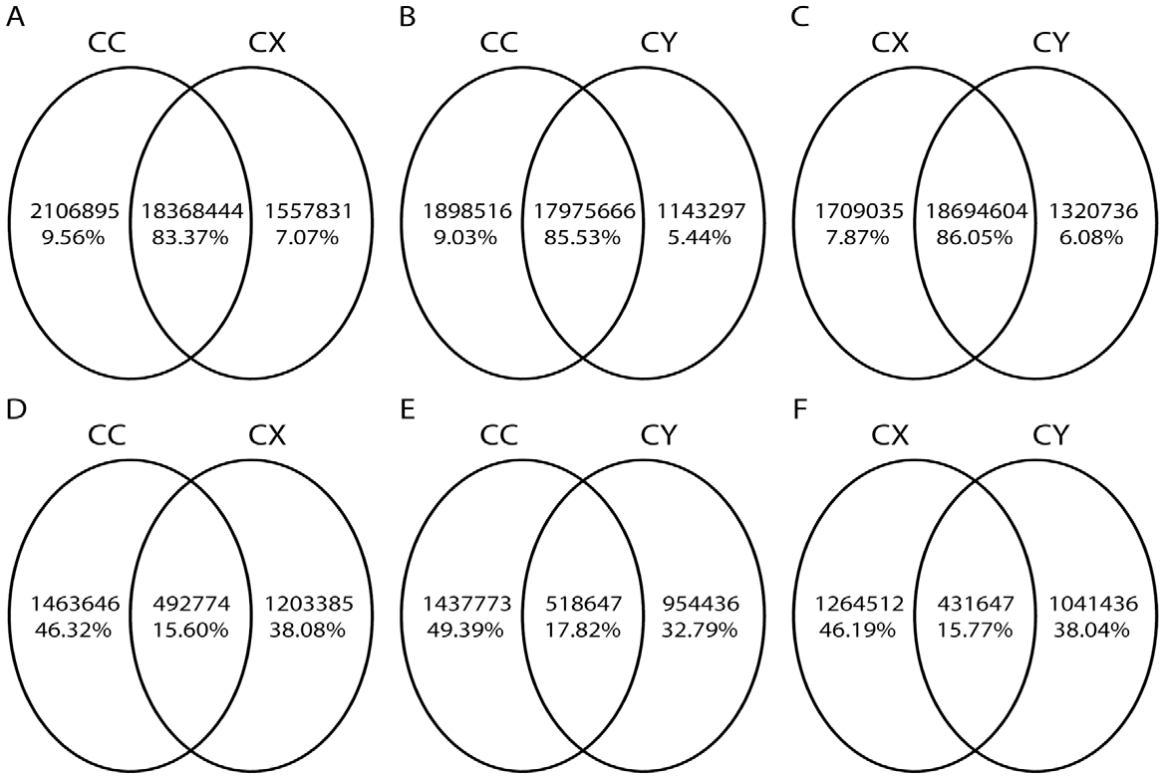
The pairwise comparisons of total small RNA reads (A, B, C) and unique small RNA reads (D, E, F) in the three libraries. The overlapping parts were their common sequences. The peculiar parts were their respective specific sequences.

Through deep sequencing small RNAs of 10 to 30 nucleotides in the three BPH small RNA libraries (CC, CY, CX), and removing the low quality sequences (low sequencing quality reads, no 3′adapter sequence reads, having 5′adapter sequence reads, no insert fragment reads, less than 18 nt reads, containing polyA reads), a total of 10.66 million, 11.37 million and 10.35 million clean reads were determined in CC, CX and CY, respectively. The length distributions of the total small RNA reads in the three libraries were shown in Figure 1, which showed a distinct bimodal distribution pattern in every library with peaks at 22 nt and 27 nt, and was similar to that of the locust and silkworm [23], [27]. The unique small RNA reads (if a small RNA has two reads or above two reads in the total small RNA reads, just leave one to exist.) were 1.956 million in CC, 1.696 million in CX and 1.473 million in CY. Through pairwise comparison of the total small RNA reads and unique small RNA reads of the three libraries, it was found out that the common sequences of total small RNA reads between any two libraries occupied a large percentage, while the library-specific reads/sequences only occupied 5% to 10% of the total small RNA reads (Figure 2). On the contrary, the common sequences of unique small RNA reads only accounted for 15% to 18%, and most of the unique small RNA reads were library-specific (Figure 2).

**Figure 3:**
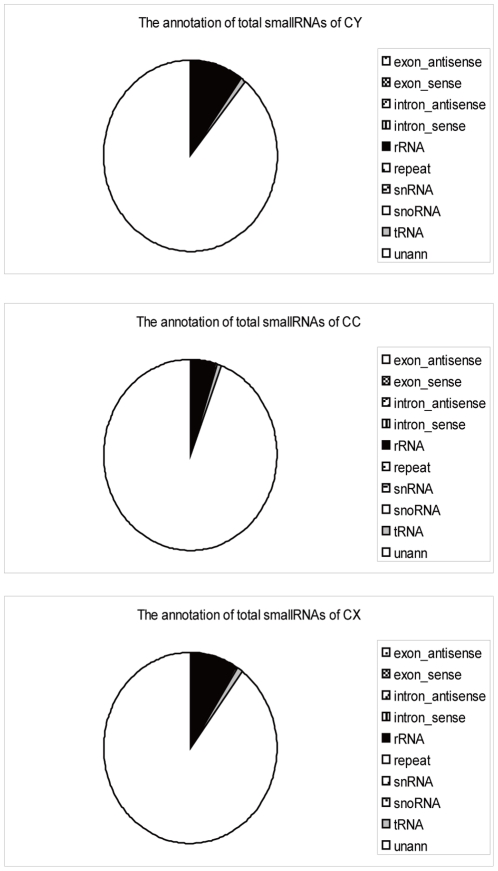
Total small RNA type annotations of the three libraries (CC, CY, CX).

From aligning the total clean small RNAs to *Drosophila melanogaster* genome (http://www.fruitfly.org/), *Drosophila melanogaster* genome repeat sequences (http://www.fruitfly.org/), Genbank (ftp://ftp.ncbi.nlm.nih.gov/genbank/) and Rfam(9.1) (http://rfam.janelia.org/), the total small RNAs can be divided into 10 categories in accordance with their arrangement priority (Figure 3). The “unann” was referred to the remaining unannotated small RNAs after the foregoing classification.

### Conserved microRNAs identification

**Figure 4:**
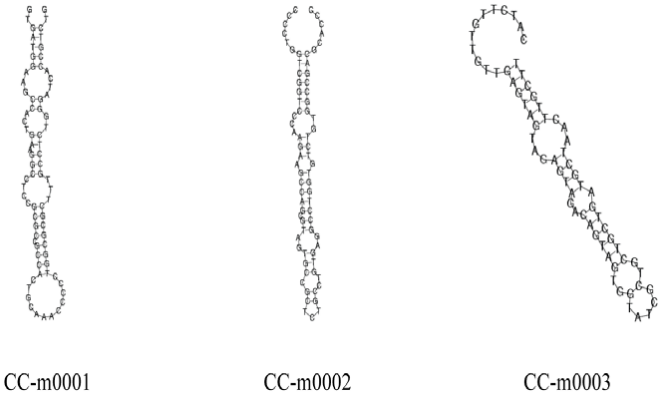
Folded structures of miRNA precursors of three conserved miRNAs in CC.

**Figure 5:**
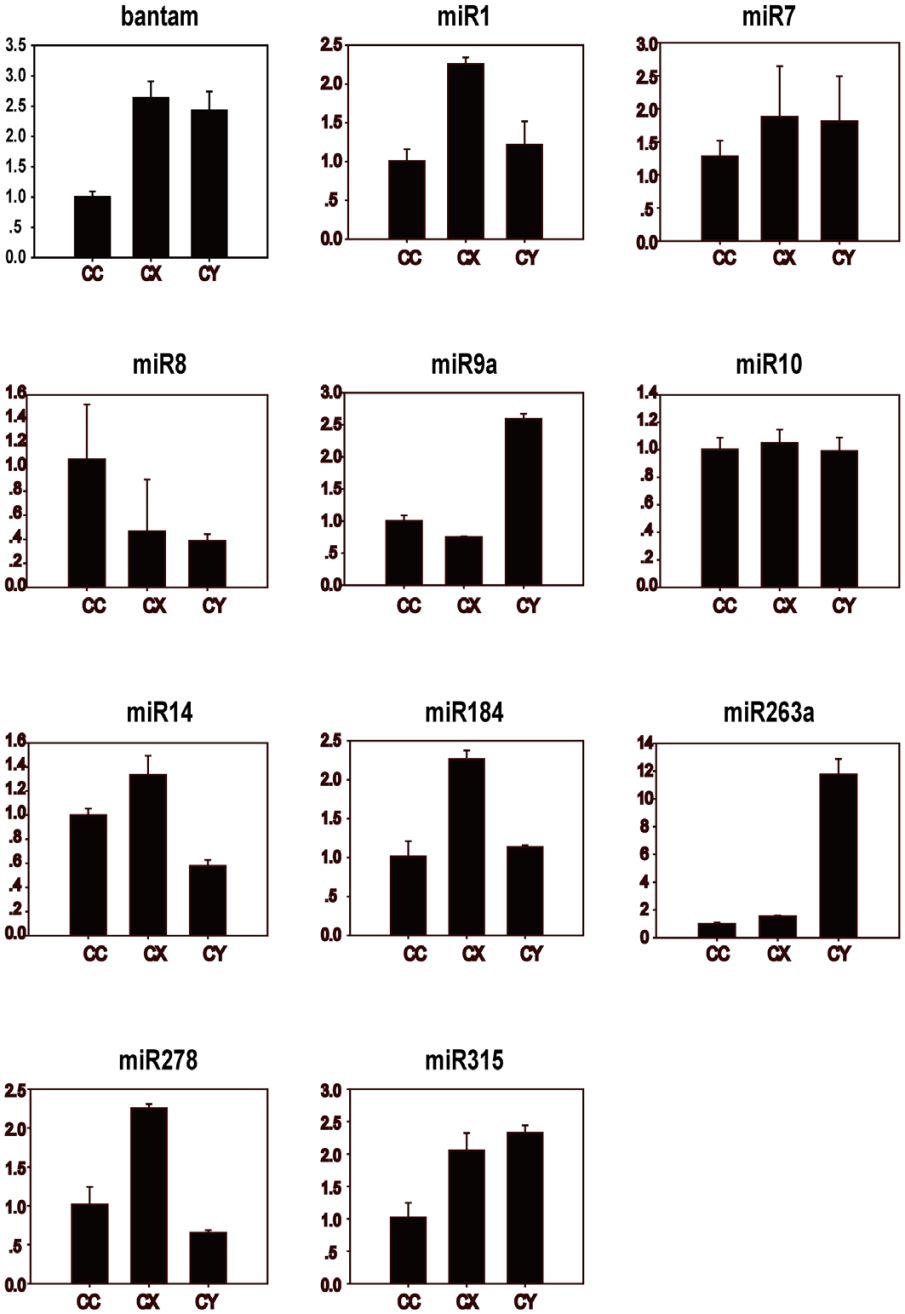
Relative quantitative PCR results of some conserved miRNAs in the three libraries (CC, CX, CY). Sample libraries were marked as abscissa and the relative expression level of the conserved miRNA as ordinate.

The remaining unannotated small RNAs were aligned with all the mature microRNAs in miRBase16.0 (http://www.mirbase.org/), which identified many conserved microRNAs according to the standards described in our Methods. The miRNA sequence with the highest expression (having the most reads) of a miRNA name was used to represent the miRNA. The expression profiles of these miRNAs are shown in Table S1, S2, and S3. Among all the known microRNAs, some come from the same microRNA family. We obtained a final list of 287, 301 and 229 miRNA family in CC, CX and CY, respectively. By aligning the representative miRNA sequences to the *Drosophila* genome and the *Nilap*arvata *lugens* integrated transcriptome (unpublished data) according to our standard, we gained 41 miRNA precursors which can fold back to a hairpin structure in CY, 40 in CC, and 42 in CX (Table S4, S5, S6, S7, S8, S9). These structures further confirmed the existence of these conserved microRNAs. Three folded structures of microRNA precursors from CC are shown in Figure 4. The failure of finding the precursors of the large part of the conserved microRNAs using our current bioinformatics method might be due to the lack of BPH genome data and the low coverage of transcriptome sequences. During this precursor finding procedure, some miRNAs that matched to the same precursor sequences with their mismatched complementary mature miRNAs were also discovered, which might be miRNA star(miRNA*) sequences(Table S4, S5, S6, S7, S8, S9). To test the existence of these mature microRNAs, relative quantitative PCR was executed for 11 conserved microRNAs including miR1, bantam, miR8 and so on (Figure 5). These conserved microRNAs existed in the three libraries, and their expression levels (reads) are somewhat different.

### Expression profiles of the known miRNAs across different developmental stage

**Figure 6:**
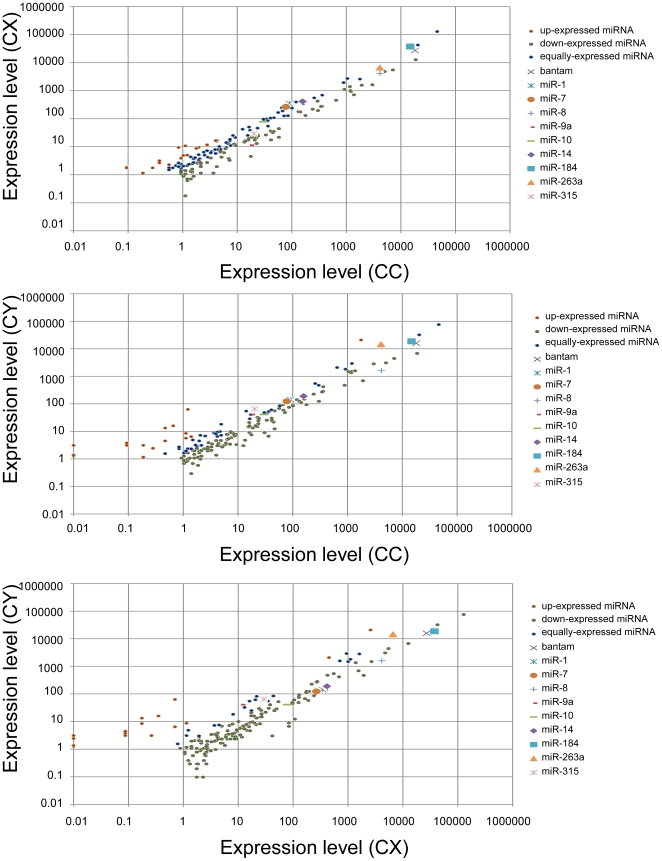
Pairwise comparison results of the expression level of all conserved microRNAs in the three libraries. Each point represents a miRNA. The X axis and Y axis show the expression level of miRNAs in two libraries, respectively. Red points represent the miRNAs with fold change of expression level between two libraries>2; Blue points represent miRNAs with 1/2<fold_change of expression level between two libraries>2; Green points represent miRNAs with fold_change of expression level between two libraries< = 1/2. These conserved miRNAs that had been detected with quantitative PCR (Figure 5) were highlighted with other different graph symbols.

**Figure 7:**
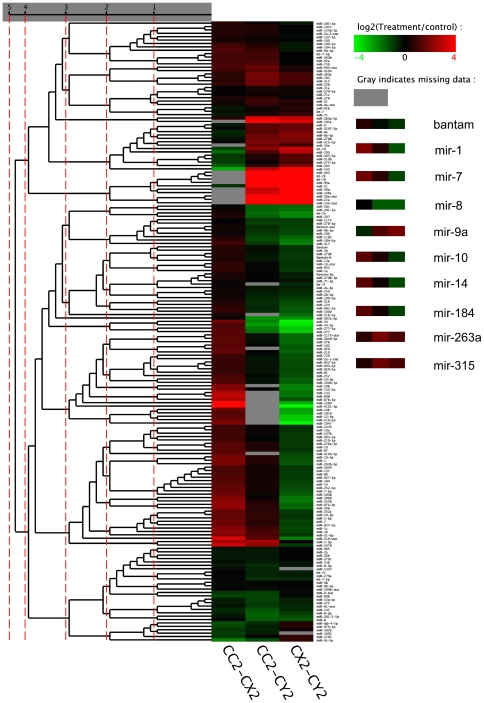
The clustering results of the conserved miRNAs based on their expression level pairwise comparison results of every two libraries. Red indicates that the miRNA has higher expression level in treatment sample, green indicates that the miRNA has higher expression in control sample and gray indicates that the miRNA has no expression in at least one sample. (CC2 represents CC; CX2 represents CX; CY2 represents CY). The expression profiles of some conserved miRNAs that had been detected with quantitative PCR (Figure 5) were listed out on the right.

The relative frequency of a miRNA as represented by the read number percentage in one library, generally represents the measurement of the miRNA expression level in the library. The expression profiles of miRNAs varied from highly specific to ubiquitous during the three developmental stages. Some miRNAs were detected ubiquitously in all the three libraries with comparable expression, while some were expressed in only one or two of the three libraries. There was also a class of miRNAs expressed in all three libraries, but those miRNAs dramatically changed their expression levels in different developmental stages. The results of the pairwise comparisons of the expression level of all the conserved microRNAs (the read number was more than 5) in the three libraries are shown in Figure 6 (detailed data is displayed in supporting Table S10, S11, S12, some conserved miRNAs that had been detected with quantitative PCR (Figure 5) were marked). Compared to the expression level in one library, the microRNA had a higher, lower, or equivalent expression level in the other library. The hierarchical clustering outcomes of all the conserved microRNAs based on the pairwise comparisons of their expression level are illustrated in Figure 7 (detailed results are displayed in Table S13, S14, S15, S16, S17) with different colors representing different expression levels. The expression profiles of some conserved miRNAs that had been detected with quantitative PCR (Figure 5) were highlighted (Figure 7). All differentially expressed miRNAs were clustered all in one cluster after five rounds of clustering. Bantam and miR1, the ordinary conserved miRNAs, had a very high abundance in every library; MiR30d was not expressed in the male adult (CX), but was present in the female adult (CC) and female larvae (CY); MiR144* and miR22a were only expressed in the female larvae (CY); MiR263a, miR9a and let7b had a higher expression in CY than in CC and CX; MiR317, miR87, miR277 and miR34 were expressed highly in CX; MiR7, miR183 and miR31 were found in CC with a lower expression than in CX and CY. These expression levels may not be entirely accurate, but can serve as a reference [28].

### Identification of new microRNAs

After obtaining the above-mentioned conserved miRNAs, the remaining sequences of the three libraries were aligned with the Drosophila genome (http://www.fruitfly.org/) and BPH integrated transcriptome(unpublished data) to forecast the novel miRNAs. Totally 71 new miRNAs in all three libraries were obtained (Table S18) using the software Mireap, after mining in the mirbase (http://www.mirbase.org/). Although the miRNA world of *D. melanogaster* has been thorough studied, the bph-m0013, matched to *D. melanogaster* genome, and was considered as the novel miRNA of *D. melanogaster*. The rest 70 new miRNAs were found from BPH integrated transcriptome. In these new miRNAs, one miRNA family containing 11 members was identified (Table 1), most of these 11 miRNAs had an identical seed region and even had an identical peripheral sequence, but their precursors were different.

**Table 1:** The 11 new miRNA members of one miRNA family in BPH.

MiRNAfamilymember	Mature sequence	Precursor sequence
bph-m0002	TCCACGAGAACCAATCAGAGA	CGTGATTAGTTCCACGAGAACCAATCAGAGAGAGAAGCCGTCTTATCACAAAGGCCTTCTCTGATTGGTTCTTG
bph-m0003	CGAGAACCAATCAGAGAAGC	TTGAATTCTTCGAGAACCAATCAGAGAAGCGTTCTTCTCACAAAAACTTTCTATGATAGGTTCTCGTCGTATTTCTC
Bph-m0005	ACGAGAACCAATCAGAGAAGC	TGCTACGAGAACCAATCAGAGAAGCATCCTTCTCAAGAAAGCCTTCTCTGATTGGTGCTTGTGACAT
Bph-m0007	CACGAGAACCAATCAGAGAAGC	TGATTAATTGCACGAGAACCAATCAGAGAAGCCGTCTTTTCAAAAACGCCTTCTCTGATTGGTTCTTGTAAAGTTAATCC
Bph-m0016	ACGAGAACCAATCAGAGAAGC	TGCTACGAGAACCAATCAGAGAAGCATCCTTCTCAAGAAAGCCTTCTCTGATTGGTGCTTGTGACAT
Bph-m0018	CACGAGAACCAATCAGAGAAG	AATAATATGTCACGAGAACCAATCAGAGAAGACATTTAAAAAAAGAAGATCTCTGATTGGTTCTCATGGCATTTAAT
Bph-m0020	ACAAGAACCAATCAGAGAAGGC	GATTAACTTTACAAGAACCAATCAGAGAAGGCGTTTTTGAAAAGACGGCTTCTCTGATTGGTTCTCGTGCAATTAATCA
Bph-m0028	ACGAGAACCAATCAGAGAAGTC	TGATTAATTCACGAGAACCAATCAGAGAAGTCTTCTTTTCTAAAAGGCATTCTCTGATTGGTTCTCATGGAGTTAATCAC
Bph-m0034	ACGAGAACCAATCAGAGAAGC	ATCAATCCTACGAGAACCAATCAGAGAAGCGTCTTTCCAAAAACGCCTTCTCTGATCGGCTCTCGAGAAACCAA
Bph-m0042	CACGAGAACCTATCAGAGAAGC	GATTAATTCCACGAGAACCTATCAGAGAAGCCGTCTTATCAAAAAGACCTTCTCTGATTGGTTCTCATGAAACCAATCA
Bph-m0052	CACGAGAACCAATCAGAGGAG	ATTAAATGCCACGAGAACCAATCAGAGGAGACTTTTTCGGAAAGAAGGCTTCTATGATTGGTTCTCGTGGAATCTAATTTAATA

**Figure 8:**
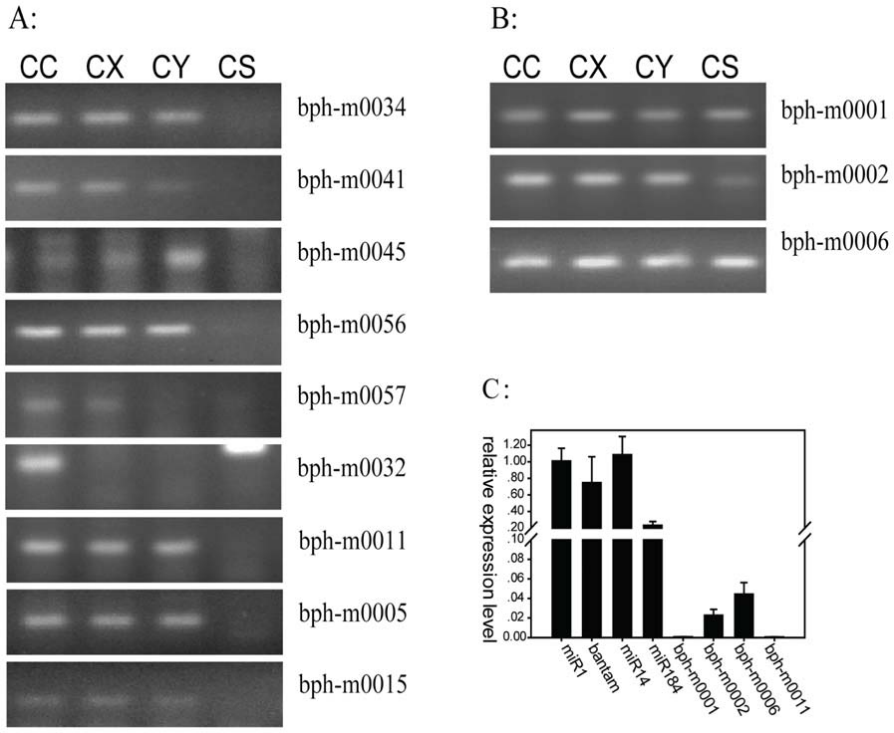
The RT-PCR results of 12 new miRNAs (in CC, CX, CY, CS (Chilo suppressalis(Walker)), and the relative expreesion level of eight miRNAs( in CC). (A) The new miRNAs which do not exist in CS. (B) The new miRNAs which do exist in all the four samples. (C) The relative quantitative expression level of four conserved miRNAs (Mir-1, bantam, Mir-14 and Mir-184) and four new miRNAs in CC (bph-m0001, bph-m0002,bph-m0006 and bph-m0011).

Like the conserved microRNAs, some of the novel microRNAs existed in all the three libraries, while some were found only in one or two of the libraries. The reverse transcription polymerase chain reaction (RT-PCR) was used to test and verify 13 novel miRNAs in CC, CX, CY and CS (Chilo suppressalis), 12 new miRNAs were amplified out in BPH (Figure 8A and 8B), among which 9 did not exist in CS (Figure 8A). With no surprise, the expression levels of the novel miRNA were generally lower compared to the conserved miRNAs [23], [27] (Figure 8C).

### Base preference of the new microRNAs of BPH

The length distributions of the known microRNAs mainly were between 18 nt to 30 nt, while the length of new miRNAs only covered 20 nt to 24 nt (Figure S1A). Base preference analysis of the front 22 nt of all the new microRNAs demonstrated that Cytosine was the least used base. The base most frequently employed was Uracil, which was the same with that of miRNAs of most species [9], [28]. Guanine most frequently appeared in the 4^th^ nt, 5^th^ nt, 17^th^ nt, 18^th^ nt, 19^th^ nt and 20^th^ nt. The twelfth base was frequently to be Uracil (data shown in Figure S1B). Analyzing the first base of the new miRNAs of every length illustrated that the first base preferences of every length miRNAs in the three libraries had large divergences, but there were also some similarities. Guanine was the least used base in the first nucleotide, and only three 22 nt-length new miRNAs used Guanine as their first nucleotide base (Figure S1C).

### Target prediction of new miRNAs

In order to predict the target genes of the novel miRNAs, we aligned the 71 new mature miRNAs to the integrated BPH transcriptome (using miRanda algorithm). Each of them aligned to ten to six hundred target sites meeting the requirements. There was also a case that one target site was targeted by several new miRNAs. The target prediction results are displayed in Table S19.

### Genome repeat sequence related small RNAs

In the small RNA alignment, there was a small amount of small RNAs that matched to the repeat sequence region of the *Drosophila* genome. Analysis results of the types of repeat sequence associated small RNAs of the three libraries were shown in Table S20. Most of the repeat sequences were rRNA, followed by transposon LTRs such as line and DNA/Tc1. Transposons, especially virus related retrotransposon, are observed in the region which is teeming with siRNA and piRNA [29], [30], [31], [32]. The relatively longer small RNAs of this part may be piRNA, while the shorter ones were siRNAs [23]. Repeat sequence can also produce other types of small RNAs [33].

### The phylogenetic evolution of conserved miRNAs

MiRNA is developing endlessly due to the biological evolution. In different species, some miRNAs are extremely specific, while others are phylogenetically conserved despite the distance of several hundred million years. Studies suggest that miRNA can serve as a marker for biological evolution [34], [35]. We divided the conserved miRNAs of BPH into several categories according to the miRNA family distributions in eight different species (Table S21). The first type was found in non-chordates, including miRNAs such as miR2, miR87 and miR252. The second type was present in the Arthropods, including miRNAs such as bantam, miR263, miR276, miR277, miR278, miR305, miR315, miR317, miR-iab-4, miR14, miR275, miR279. The third category contained miRNA both from some of chordates and non-chordates, including miRNAs such as miR125, miR133, miR184, miR210, miR137 and miR31. The fourth type containing miRNAs that have been detected in all the chordates and non-chordates belongs to the ancient group, for example miR1, miR124, miR9, let7 and miR34. Without any doubt, the new miRNA of BPH reported in this manuscript was the last category representing BPH or Delphacidae.

## Discussion

1. Pairwise comparisons of unique small RNA reads and total small RNA reads of the three libraries showed that the common sequences of total small RNA reads comparison of every two libraries accounted for more than 80%, while the common sequences of unique small RNA reads comparison of every two libraries occupied only 20%, this may indicated that the regulation of small RNAs in the whole BPH body shared many similarities regardless of the development stages, and when they are at individual developmental stages, the discrepant small RNAs could be produced for the respective physiological and metabolic activity regulation. MiRNA could be one of the senior regulators.

2. Our study demonstrated that there is a rich small RNA world in BPH. This is the first achievement to investigate the whole range of small RNAs of BPH, which possess their own characteristics. The length distribution manifested certain regularity (Figure 1) among organisms like the locust and silkworm. We had verified that feeding BPH with dsRNA in vitro can lead to the decreased expression of homologous genes in BPH [25], [26], however, the requisite dsRNA concentration is very high (unpublished results). How does the RNAi mechanism of BPH work? What are the features of its small RNAs? How can we design the dsRNA for gene interference in BPH? There are still lots of such unsolved questions. Our discoveries of all the small RNAs of BPH provided pivotal clues to the utilization of RNAi to resist BPH.

3. We have found many animal conserved miRNA families in the three developmental stage libraries. The various functions of these miRNAs may correlate closely with the evolution of the organism's body plan as well as phenotypic variation within related species. It is crucial to understand the conservation of miRNAs in a broader set of insects in order to understand the function and evolution of miRNA in animals [20]. There will be more birth and death of miRNA families to go along with the endless biology evolution [34], [35]. MiR87 and miR2 were only found in non-chordates, so they may be produced with non-chordates; Bantam,miR263 and miR276 existed only in insect, so they may be produced with arthropod insects. BPH is a hemimetabolous insect, and is opposite to silkworm, which is a holometabolous insect. Our miRNA family analysis of eight species demonstrated that the miRNA repertoire of BPH was more similar to that of fruit fly than silkworm. Here, we only analyzed the small RNAs in three developmental stages, more novel and useful small RNAs could be detected with more different developmental stages.

4. MiRNAs display a phylogenetic conservation on both structure and sequence [9], [28], [36], [37], which indicates the importance of the function of miRNAs [38]. *Drosophila melanogaster* is the prototype for genetic analysis of arthropods [20] and the miRNA world has been intensively studied [34], [39], [40]. In our study, some functional miRNAs of *Drosophila* also have congeners in BPH and have their own special expression profiles in the three developmental stages (see Figure 5). Bantam, targeting the *Mei-p26* gene, controlled the growth rate of normal tissue by regulating cell growth, cell division and apoptosis in *Drosophila*, and the *Bantam*-mutant animals were smaller than the wild type, owing to a reduction in cell number but not in cell size [41], [42]. Bantam was highly expressed in all the three BPH libraries and the lowest level were shown in CC amongst. In *Drosophila*, miR14 was needed for fat metabolism inhibits cell death, and the *miR14*-mutant fruit fly was pressure-sensitive and had shortened life expectancy. In *D. melanogaster*, miR14 was observed in all life stages and displayed a strong starting expression signal from late embryonic to adult stages in Asian malaria mosquito [40]. In our research, miR14 had a higher abundance in CC and CX than in CY. *MiR8*-mutant *Drosophila* accelerated neurasthenia, directly inhibited the *wntless* gene and led to behavioral defects, which resulted in a lower survival rate and lays abnormal eggs. In addition to the expression signals in the brain, wing discs, and leg discs, miR8 was also strongly noticed in the fat body of *Drosophila* [43], [44]. BPH miR8 was minimally expressed in CY and CX, but was highly expressed in CC. MiR9a which regulates the 3′-UTR of the *senseless* gene can mediate accurate differentiation of sensory organs in *Drosophila*. *MiR9a* mutations can led to a significant reduction of wing tissues from increased feeling setaes and wing primordium's apoptosis [45], [46]. MiR9a had a lower expression level in CX and CC than in CY of BPH, which was consistent with what was observed in fruit fly and Asian malaria mosquito [21]. In *Drosophila*, miR1 specifically expressed in the mesoderm and its muscle cell derivatives, played an important role in the development of muscles and the heart, and regulateed Notch signaling [47], [48]. In BPH, miR1 revealed a much higher abundance in CX than in CC and CY. *MiR10* is located in the antennae gene complex in fruit fly, and one target of miR10 is the *sex combs reduced* gene [40]. BPH miR10 was expressed the same in all the three libraries of BPH. MiR7 which regulated the Notch signaling pathway and ensured the accurate differentiation of sense organs in *Drosophila* [49], was expressed relative highly in CX and CY of BPH. MiR263a/b maintained robustness during the development of sensory organs through protecting sensory organs against wilt. MiR263a/b of *Drosophila* are expressed in sense organ precursors in the embryo and in the mechanosensory organs of the eyes, antennae and haltere [50]. MiR263a was expressed much greater in CY of BPH compared to the level in CC and CX. MiR184 is expressed in the embryos, nymphs and adults of *Drosophila*, with a significant increase of the expression level during embryo development [51]. MiR184 was expressed in all the three libraries, with the highest expression in CX, and the expression level difference between CX and CC or CY was remarkable. The mutant of *miR278* in *Drosophila* can lead to defective energy homeostasis and relatively small insulin production [52]. MiR278 had low expression level in all the three libraries of BPH and CX got the highest expression level of the three libraries. MiR315 is a potent activator of wingless signaling in fruit fly [53], it had a higher expression level in CY than in CC and CX of BPH.

Our three small RNA libraries represented three developmental stages (female adult, male adult and last instar female nymph). Most of the miRNAs homoplastically come from all the three developmental stages, and some miRNAs had very distinct expression patterns in the three libraries. Wg/Wnt signaling was highly regulated in insecta, regulating the development of the wing through precisely controlling the associated signaling. Inappropriate activation or inhibition of Wg/Wnt pathway results in developmental defects and diseases in silkworm [27], such as when nymphs growing into adults. We speculated that miR8, miR9a, miR315 may execute the similar important functions in the wing development between BPH and the fruit fly through their expression profiles in BPH. Reproduction is also a complex physiological activity. Adult libraries may reveal some information about reproduction [27]. We have found that miR30d was not expressed in the male adult (CX), but was present in the female adult (CC) and female larvae (CY). MiR317, miR14, miR184, miR277 and miR34 were expressed greater in CX than in CC and CY. The expression profiles of these miRNAs indicated that they may be male-associated miRNAs. During the reproduction process, other small RNA molecules, called piRNAs, performed their important function of defending the genome against selfish DNA elements such as the transposons in germline cells. Taken together, all the information we have obtained may offer significant assistance in discovering more about BPH.

5. The generation mechanisms of siRNA and piRNA in animals were not as clearly understood as that of miRNA [36], but the existence of siRNA and piRNA in organisms is a indisputable reality, and their generation mechanisms will become increasingly clear [15], [16], [54]. PiRNA is a long single strand small RNA and regulates transposons or other physical activities in germline cells. SiRNA is about 19 nt to 24 nt in length, it is a double strand small RNA, and plays an important role in virus defense and transposon regulation [23], [32]. Some repeat sequences related small RNAs with a length of about 22 nt may represent siRNAs, and that with a length of about 27 nt may represent piRNAs in all three stages. Due to the distant relationship between fruit fly and BPH, these kinds of small RNAs that we have obtained were little. There are certainly a lot of siRNAs and piRNAs existing in our libraries.

6. We may discovered 70 (except the one aligned to *Drosophila* genome) novel miRNAs in the three developmental stages of BPH (CC, CY, CX). Highly specific miRNAs may have exclusive functions in particular organisms and are possibly involved in the regulation of lineage specific pathways. Comparing all the newly identified miRNAs to the animal microbase (http://www.mirbase.org/), there were no matches for any miRNA, and via the confirmation of the existence of several new miRNAs by reverse transcription polymerase chain reaction (RT-PCR) (Figure 8A and 8B), they were considered as authentically novel miRNAs. Base distribution at each location of these new miRNAs also revealed certain preferences (Figure S1A and S1B). The new miRNAs were expressed at low abundances, which was consistent with the previous research results [23], [27]. There may be some authentic correlation between evolutionary conservation of miRNAs and their expression levels. After realigning these new miRNAs to the intergrated transcriptome (in order to look for their target genes), many targets on the transcriptomes were found for all the new miRNAs.

### Conclusion

We have constructed three developmental stage small RNA libraries of BPH, where we detected a lot of small RNAs. Many conserved miRNA families have been identified and we also have predicted some novel miRNAs. All the expression profiles of these miRNAs in the three libraries have been analyzed. Some miRNAs displayed significantly different expression patterns among the three stages, which indicated they may play important roles in regulating physiology and shape differences of BPH and can be used to control the growth and development of BPH. Some miRNA* have been identified and the existence of their mature sequences were further confirmed. MiRNA family evolution analysis showed that the evolution process of BPH miRNA was similar to that of other species, and miRNAs can be deemed as a sign of species. In addition to miRNAs, there were many other types of small RNAs in BPH, such as repeat sequence related small RNAs. The discovery of all these small RNAs in BPH provided an important basis for recognizing and defending BPH. The small RNA world and the RNAi mechanism of BPH will be clearer if the genome sequence of BPH is made available.

## Materials and Methods

### Preparation of three developmental stage samples and the total RNA extraction

This study constructed three BPH small RNA libraries for three developmental stages. They were the female adult (CC, three days after emergence), male adult (CX, three days after emergence), and female nymph (CY, the fifth instar nymph). The CC sample and CX sample each were composed of 20 insects with a similar scale. The CY sample was composed of 30 insects with a similar scale. All the samples come from Huazhong Agriculture University (which we fed in our lab). Total RNA of the whole animals of the three samples was extracted using TRIzol reagent (Invitrogen, Carlsbad, CA, USA). The extracted RNA was sent to Beijing Genomics Institute (BGI, Shenzhen, China) to test their integrity.

### Small RNA library construction and deep sequencing

Small RNAs that were less than 30 nt were collected through gel separation, afterwards, these small RNAs were ligated with a 5′adaptor and 3′adaptor to its ends and purified (T4 RNA Ligase, 200 U, 30 U/ul, Takara:D2050); Then these small RNAs were reverse transcribed with the complimentary sequence of the 3′ adaptor (SuperScript® III Reverse Transcriptase, Invitrogen:18064-014), and then the adaptor sequence primers were used to amplify the reverse transcription products (PCR Thermocycler PTC-100, BioRad). The PCR amplification products were sequenced by BGI using solexa sequencing technology after purification.

### The small RNA annotation

A large quantity of clean small RNAs were obtained after the raw sequences processing. Then we targeted these small RNAs to the Drosophila genome using SOAP software, and analyzed their distribution. The remaining small RNAs were compared with Drosophila genome repeat sequences using the tag2repeat software (BGI) to find out the repeat sequence related small RNAs. The residual small RNAs were then compared to the Genbank and Rfam (9.1) database to find out the possible rRNA, scRNA, snoRNA, snRNA and tRNA. Finally the small RNAs were annotated in accordance with the priority order (exon = intron >rRNA>repeat sequence>scRNA>snoRNA>snRNA>tRNA ; Genbank>Rfam).

### The integrated BPH transcriptome

There were a total of 88194 contigs (>100 bp, mean length was 228) and 76406 unigenes (>100 bp, mean length 293 bp) in our transcriptome. The number of the annotated sequences was 26228.

### Identification of conserved miRNAs

The remaining small RNAs (after clustering) were compared with all the mature animal miRNA and miRNA precursor sequences of miRBase16.0 (BGI: Tag2miRNA; parameters: two mismatches and gap< = 2 bp were allowed to exist around the seed region), consequently, the conserved miRNA were obtained. The mature miRNA having the highest expression of a miRNA name was used to instead of the miRNA. The representative mature miRNA sequence of a miRNA was then aligned to the Drosophila genome and the integrated BPH transcriptome to find its precursor using Mireap (BGI) (using its default parameters).

### Prediction of new miRNAs

The remaining sequences after conserved miRNAs indentification were aligned to the Drosophila genome and the integrated BPH transcriptome in order to identify new BPH miRNAs. Certain target sequences around the small RNA were used to explore the secondary structure and folding energy (−18 kcal/mol). (BGI:Mireap) (http://sourceforge.net/projects/mireap/)

### Quantitative RT-PCR assay of conserved miRNAs and RT-PCR assay of new miRNAs

First, reverse transcription reactions were conducted for mature miRNAs and control actin. Reverse transcription reaction system (20 ul) contained 1 ug RNA samples (after DNAseI treated), 50 nM stem–loop RT primer (50 nt, the frontal 44 nt were the stem-loop region, they were stationary; the last 6 nt were reverse complementary to the 3′portion of the miRNA moleculars) or 20 nt actin primer (GATGTCACGCACGATTTCAC it was reverse complementary to the actin gene of BPH), 5*RT buffer (P/N:y02321, Invitrogen), 0.25 mM dNTPs (MD0120, Pharmacia), 0.5 ul SuperScript® III Reverse Transcriptase (P/N: 56575, Invitrogen), 0.5 ul 0.1*DTT (P/N: Y00147, Invitrogen) and DEPC treated water. The 20 ul reactions were incubated in an Applied Biosystems 9700 Thermocycler for 40 min at 50°C, 5 min at 85°C and than held at 4°C. Second, the reverse transcription reaction products was used to for RT-PCR (9700 Thermocycler, Applied Biosystems; Takara taq™: DR100D, Takara) and relative quantitative RT-PCR (7500 Thermocycler, Applied Biosystems; SYBR^R^ *Premix* Ex Taq: DRR041A, Takara). The primers for the RT-PCR and relative quantitative RT-PCR of miRNAs all contain universal primer (GTGCAGGGTCCGAGGT, the loop region of forward stem–loop RT primer) and miRNA specific primers (21 nt, 6 nt of the 5′portion were stationary(CGCAGC)), next 15 nt were the 5′portion sequence of the miRNAs). The actin gene was used as reference gene of RT-PCR and relative quantitative RT-PCR (PCR primer:F-CCAACCGTGAGAAGATGACC; R-GATGTCACGCACGATTTCAC).

### Target gene prediction of new miRNA

The new miRNAs were aligned to the integrated BPH transcriptome to predict the target genes by miRanda algorithm [55] (with parameters: -sc 150, -en 30).

## Supporting Information

Figure S1 **The Characteristics of all the new miRNAs of BPH.** (A) The length distribution of all the new miRNAs. (B) The nucleotide base bias at each position of front 22 nucleotides of all the new miRNAs. (C) The base bias of the first nucleotide of every length new miRNAs of BPH.
(TIF)

Table S1 **The conserved miRNAs and their expression level in CC.**
(XLS)

Table S2 **The conserved miRNAs and their expression level in CX.**
(XLS)

Table S3 **The conserved miRNAs and their expression level in CY.**
(XLS)

Table S4 **The precursor predicted results of some CC conserved miRNAs that aligned to** ***Drosophila melanogaster*** **genome.**
(XLS)

Table S5 **The precursor predicted results of some CC conserved miRNAs that aligned to BPH transcriptome.**
(XLS)

Table S6 **The precursor predicted results of some CX conserved miRNAs that aligned to** ***Drosophila melanogaster***
(XLS)

Table S7 **The precursor predicted results of some CX conserved miRNAs that aligned to BPH transcriptome.**
(XLS)

Table S8 **The precursor predicted results of some CY conserved miRNAs that aligned to** ***Drosophila melanogaster*** **genome.**
(XLS)

Table S9 **The precursor predicted results of some CY conserved miRNAs that aligned to BPH transcriptome.**
(XLS)

Table S10 **The expression level pairwise comparison results of the conserved miRNAs(>5 reads) in CC and CX.** Normalization forum∶Normalized expression = Actual miRNA count/Total count of clean reads*1000000. Fold-change formula∶Fold change = log2 (treatment/control).
(XLS)

Table S11 **The expression level pairwise comparison results of the conserved miRNAs(>5 reads) in CC and CY.**
(XLS)

Table S12 **The expression level pairwise comparison results of the conserved miRNAs(>5 reads) in CY and CX.**
(XLS)

Table S13 **The first clustering results of all the conserved miRNAs based on the pairwise comparison results.**
(XLS)

Table S14 **The second clustering results based on the first clustering results of all the conserved miRNAs.**
(XLS)

Table S15 **The third clustering results based on the second clustering results of all the conserved miRNAs.**
(XLS)

Table S16 **The fourth clustering results based on the third clustering results of all the conserved miRNAs.**
(XLS)

Table S17 **The fifth clustering results based on the third clustering results of all the conserved miRNAs.**
(XLS)

Table S18 **The new miRNAs and their precursors in all the three libraries.**
(XLS)

Table S19 **The target gene prediction results of all the new miRNAs.**
(XLS)

Table S20 **The small RNA associated repeat sequence types in CC, CX and CY.**
(XLS)

Table S21 **The distributions of the conserved miRNA families in eight species.**
(XLS)
