# Supplementary figures and images for: Characterization and Comparative Analysis of Small RNAs in Three Small RNA Libraries of the Brown Planthopper (Nilaparvata lugens)
Source: PLoS One. 2012 Mar 6;7(3):e32860. doi: 10.1371/journal.pone.0032860 (PMC3295781; doi:10.1371/journal.pone.0032860)

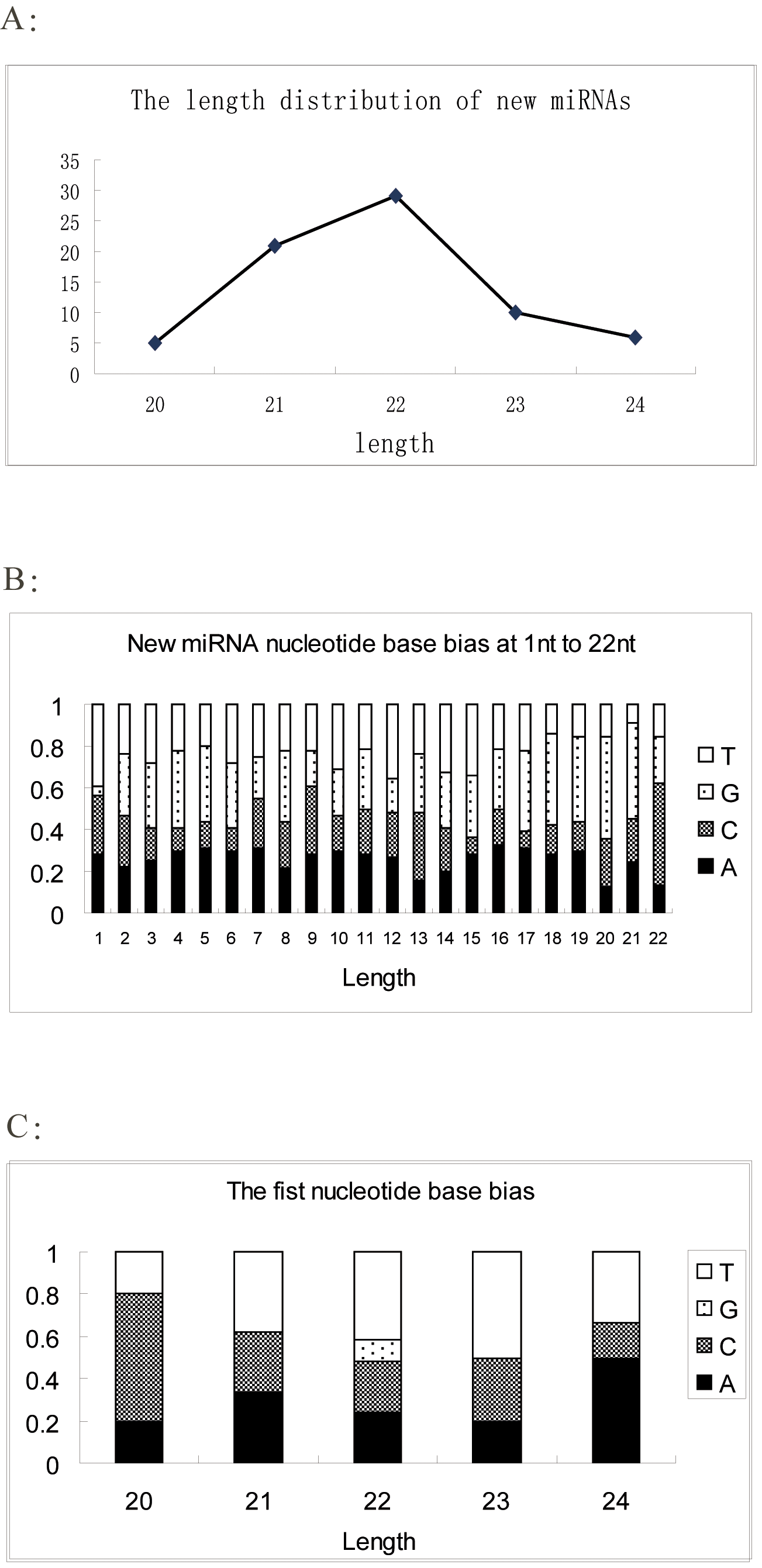

Supplement: Figure S1 — The Characteristics of all the new miRNAs of BPH. (A) The length distribution of all the new miRNAs. (B) The nucleotide base bias at each position of front 22 nucleotides of all the new miRNAs. (C) The base bias of the first nucleotide of every length new miRNAs of BPH. (TIF) [file pone.0032860.s001.tif]
